# Supplementary material for: Causal Relationship between Aging and Anorexia Nervosa: A White-Matter-Microstructure-Mediated Mendelian Randomization Analysis
Source: Biomedicines. 2024 Aug 16;12(8):1874. doi: 10.3390/biomedicines12081874 (PMC11351342; doi:10.3390/biomedicines12081874)
Supplement: Supplementary file 1 [file biomedicines-12-01874-s001.zip › Supplementary Figure/Figure S4/Singe SNP plot of MR analysis for TL to ubm-b-1906.pdf]

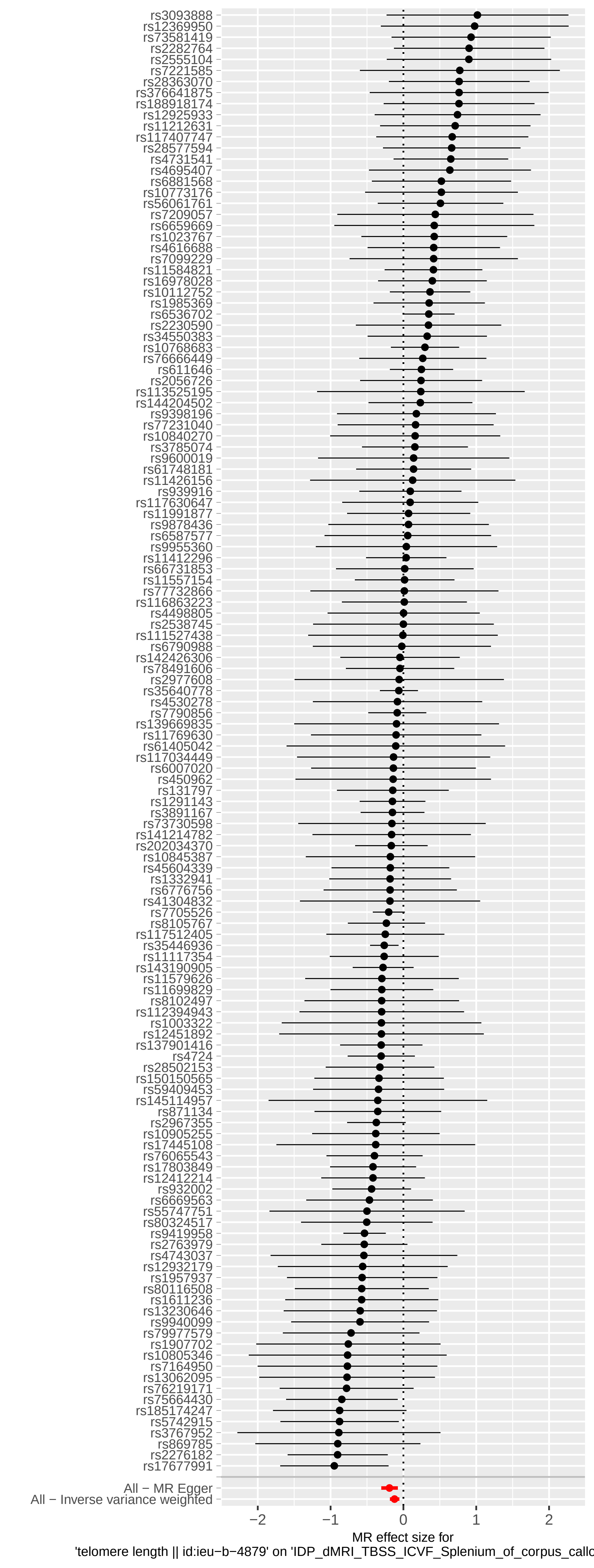
rs3093888
rs12369950
rs73581419
rs2282764
rs2555104
rs7221585
rs28363070
rs376641875
rs188918174
rs12925933
rs11212631
rs117407747
rs28577594
rs4731541
rs4695407
rs6881568
rs10773176
rs56061761
rs7209057
rs6659669
rs1023767
rs4616688
rs7099229
rs11584821
rs16978028
rs10112752
rs1985369
rs6536702
rs2230590
rs34550383
rs10768683
rs76666449
rs611646
rs2056726
rs113525195
rs144204502
rs9398196
rs77231040
rs10840270
rs3785074
rs9600019
rs61748181
rs11426156
rs939916
rs117630647
rs11991877
rs9878436
rs6587577
rs9955360
rs11412296
rs66731853
rs11557154
rs77732866
rs116863223
rs4498805
rs2538745
rs111527438
rs6790988
rs142426306
rs78491606
rs2977608
rs35640778
rs4530278
rs7790856
rs139669835
rs11769630
rs61405042
rs117034449
rs6007020
rs450962
rs131797
rs1291143
rs3891167
rs73730598
rs141214782
rs202034370
rs10845387
rs45604339
rs1332941
rs6776756
rs41304832
rs7705526
rs8105767
rs117512405
rs35446936
rs11117354
rs143190905
rs11579626
rs11699829
rs8102497
rs112394943
rs1003322
rs12451892
rs137901416
rs4724
rs28502153
rs150150565
rs59409453
rs145114957
rs871134
rs2967355
rs10905255
rs17445108
rs76065543
rs17803849
rs12412214
rs932002
rs6669563
rs55747751
rs80324517
rs9419958
rs2763979
rs4743037
rs12932179
rs1957937
rs80116508
rs1611236
rs13230646
rs9940099
rs79977579
rs1907702
rs10805346
rs7164950
rs13062095
rs76219171
rs75664430
rs185174247
rs5742915
rs3767952
rs869785
rs2276182
rs17677991
All − MR Egger
All − Inverse variance weighted
−2
−1
0
1
2
MR effect size for
'telomere length || id:ieu−b−4879' on 'IDP_dMRI_TBSS_ICVF_Splenium_of_corpus_callo
